# Impact of advanced paternal age on reproductive outcomes in preimplantation genetic testing cycles of young female: a retrospective cohort study

**DOI:** 10.3389/frph.2025.1750842

**Published:** 2026-01-23

**Authors:** Nana Kong, Min Li, Aiming Wang, Bin Liu, Yuliang Shen, Qingao Xu, Xina Wang, Zhuqing Ji, Xueying Yu, Wei Shang, Weizhou Wang, Yong Zhao

**Affiliations:** 1 Senior Department of Obstetrics and Gynecology, the Seventh Medical Center of Chinese PLA General Hospital, Beijing, China; 2 Center for Assisted Reproductive Medicine, Department of Obstetrics and Gynecology, the Sixth Medical Center of PLA General Hospital, Beijing, China

**Keywords:** advanced paternal age, clinical pregnancy, DFI, high-quality blastocyst formation, PGT-A

## Abstract

**Introduction:**

Although advanced paternal age (APA) is increasingly scrutinized in reproductive medicine, its independent impact on embryo development and clinical outcomes remains contentious, particularly when controlling for maternal age and embryo ploidy.

**Methods:**

This retrospective cohort study analyzed 357 preimplantation genetic testing for aneuploidy (PGT-A) cycles from couples with non-advanced maternal age (≤35 years). Cycles were stratified by paternal age into three groups: <35, 35–39, and ≥40 years. We compared sperm DNA fragmentation index (DFI), embryo development metrics, and clinical outcomes across these groups.

**Results:**

Men aged ≥40 years exhibited significantly higher levels of sperm DFI compared to both younger groups (both *P* < 0.05). While no significant differences were observed in normal fertilization, high-quality embryo rates, or euploid blastocyst rates across paternal age groups, blastocyst development was notably impaired in the APA group. Specifically, the ≥40-year group demonstrated significantly reduced blastocyst formation rates (57.3% vs. 68.6% and 67.2%) and high-quality blastocyst formation rates (33.2% vs. 41.3% and 40.2%) compared to the <35 and 35–39 groups, respectively. Crucially, multivariate regression analysis identified DFI as an independent factor, with higher DFI significantly associated with a reduced likelihood of forming high-quality blastocysts (OR = 0.987, *P* = 0.046) and achieving a clinical pregnancy (OR = 0.961, *P* = 0.036). The sensitivity analysis demonstrates that even when examining a population of very young women (≤32 years) where the influence of maternal age on oocyte quality is expected to be minimal and uniform, the negative association between sperm DFI and embryo development potential persists (aOR = 0.980, *P* = 0.009).

**Conclusion:**

Our findings indicate that APA itself does not directly affect blastocyst ploidy status but is associated with significantly elevated sperm DNA fragmentation. Despite the lack of direct evidence, the detrimental effects of APA on high-quality blastocyst formation and clinical pregnancy rates are probably associated with this increase in DFI. This study underscores the critical role of sperm DNA integrity in reproductive success and suggests that DFI assessment should be considered in the clinical evaluation of older men undergoing infertility treatments.

## Introduction

In recent decades, the average age at which couples initiate reproduction has risen significantly, with the mean age now approximating 30 years in numerous countries (1). This demographic shift is attributed to increased life expectancy, evolving societal expectations, and the postponement of marriage, all of which contribute to the trend of delayed parenthood. The advent and enhanced accessibility of assisted reproductive technology (ART) have provided older couples with greater opportunities to conceive, thereby elevating the average paternal age at first childbirth. While the detrimental effects of advanced maternal age on reproductive outcomes and offspring health are well-documented, the impact of paternal age on sperm quality and pregnancy outcomes remains ambiguous (2, 3).

A survey encompassing 1976 women, controlling for maternal age, revealed a reduced pregnancy rate (52.9% vs. 76.8%) among couples with advanced paternal age (≥45 years) compared to those with younger paternal age (<25 years) (4). Investigations into intracytoplasmic sperm injection (ICSI) treatment efficacy have yielded conflicting perspectives regarding the correlation between paternal age, semen quality, and embryological and clinical outcomes. While some studies associate advanced paternal age (APA) with diminished sperm quality and poorer clinical outcomes (5, 6), others report no significant link between age and clinical parameters (7) or sperm metrics (8, 9). The conflicting findings in existing studies may be due to the challenge of separating paternal and maternal influences on reproduction (10).

It is important to note that most of these findings are from ICSI or oocyte donation cycles, however, oocyte donor models may not be sufficient to assess male contribution to fertility because younger oocytes may compensate for the reproductive potential of low quality sperm. Data on the effect of paternal age on cycles of preimplantation genetic testing for aneuploidy (PGT-A) are limited and inconclusive although PGT-A can control the negative effects of advanced maternal age on embryo ploidy and quality.

This study aims to elucidate the potential effects of APA on embryo development and clinical outcomes within a PGT-A framework at the Reproductive Medicine Center, Sixth Medical Center of Chinese PLA General Hospital (Beijing, China). Employing stringent inclusion and exclusion criteria, this retrospective cohort study focused on couples with maternal age ≤35 years, normal karyotypes, and a controlled body mass index (BMI) range of 18.5 kg/m^2^–25 kg/m^2^ for enrolled women. Analyzed variables encompassed IVF laboratory outcomes—such as fertilization rates, high-quality embryo rates, blastocyst formation rates, and high-quality blastocyst formation rates—as well as clinical outcomes, including clinical pregnancy rate (CPRs), live birth rate (LBRs), and miscarriage rates (MRs), among others.

## Patients and methods

### Patients

This retrospective cohort study, conducted at the Reproductive Medicine Center of the Sixth Medical Center, Chinese PLA General Hospital, analyzed data from all PGT-A treatment cycles using fresh spermatozoa between January 2018 and October 2024, excluding repeat cycles from the same couple to ensure data independence. To minimize sampling bias, rigorous quality control was applied to ART data, with inclusion criteria restricted to female patients aged ≤35 years and BMI 18.5–25 kg/m^2^, prioritizing single euploid blastocyst transfers with optimal morphological grading. Participants were stratified by paternal age into three subgroups (<35, 35–39, and ≥40 years), while exclusions comprised donor sperm use, female factors (uterine malformations, infections, endometriosis, PCOS, or autoimmune disorders), male factors (BMI >30 kg/m^2^ or severe oligo-astheno-teratozoospermia) and couples with genetic diseases. Lifestyle factors, including smoking status and alcohol consumption, were assessed for both partners via a standardized self-report questionnaire and confirmed during a structured clinical interview prior to treatment. Only non-smokers and patients without a history of excessive alcohol use were included. The study was approved by the Ethics Committee of the Sixth Medical Center, PLA General Hospital (HZKY-PJ-2021-24), with written informed consent obtained from all participants.

### Semen analysis and preparation

Semen samples were collected via masturbation into a sterile container following 2–7 days of ejaculatory abstinence on the day of oocyte retrieval. After complete liquefaction (30–60 min), the samples were analyzed for volume, concentration, motility, and morphology in accordance with the WHO 2010 guidelines (5th edition) (11), and the results were recorded. The sperm morphology evaluation was stained with Diff-Quik and performed by the Kruger Strict. All semen were washed by density gradient centrifugation, preparation for ICSI.

### Assessment of sperm DNA fragmentation index (DFI)

Sperm DNA fragmentation was evaluated using the Sperm Chromatin Structure Assay (SCSA). Briefly, diluted semen samples were treated with a mild acid detergent followed by acridine orange (AO) staining. The samples were then analyzed using BD FACS Canto II ﬂow cytometer (Becton Dickinson, Franklin Lakes, NJ, USA). A minimum of 10,000 events were acquired per sample. DFI was calculated as the percentage of sperm with denatured DNA using SCSAsoft® analysis software. A DFI value of ≥25% was defined as high fragmentation, based on the established clinical threshold. For quality control, a reference sample was included in each run. The intra- and inter-assay coefficients of variation in our laboratory were <5% and <8%, respectively. All samples were analyzed in a single run unless the initial analysis failed technical quality checks.

### ICSI and laboratory procedures

All couples undergoing ICSI were prospectively enrolled for PGT-A based on established clinical indications. Approximately 1–2 h after oocyte retrieval, cumulus cells were removed via enzymatic digestion using 80 IU/mL hyaluronidase solution (Fujifilm, Japan) to expose the first polar body. ICSI was performed by an experienced embryologist following ≥1 h of oocyte incubation. Post-injection, oocytes were cultured in G-1 Plus medium (Vitrolife, Sweden) under mineral oil at 37 °C in a tri-gas incubator (5% O_2_, 6% CO_2_). Fertilization was assessed 17 ± 1 h post-injection, followed by cleavage evaluation at 44 ± 1 h. On day 3, embryos were transferred to G-2 Plus medium (Vitrolife, Sweden) for extended culture through day 6 to support blastocyst development.

### Outcomes

The primary outcomes included the blastocyst formation rate (defined as the number of blastocysts obtained on day 5 or day 6 divided by the number of normally fertilized oocytes), the high-quality blastocyst formation rate (defined as the number of high-quality blastocysts obtained on day 5 or day 6 divided by the number of normally fertilized oocytes), the euploid blastocyst rate (defined as the number of euploid blastocysts divided by the total number of blastocysts), and the mosaicism rate (defined as the number of mosaicism blastocysts divided by the total number of blastocysts). Secondary outcomes included the normal fertilization rate [defined as the number of normally fertilized oocytes divided by the number of injected metaphase II (MII) oocytes] and the high-quality embryo rate (defined as the number of high-quality embryos on day 3 divided by the number of normally fertilized oocytes). Cleavage-stage embryos were assessed and graded on day 3 according to Cutting's criteria (12), which evaluated blastomere number, cell symmetry, and fragmentation. Grade I–II embryos were defined as high-quality embryos, and they met the following criteria: 6–8 cells on day 3 post-fertilization, homogeneous blastomeres, and fragmentation <10%. Blastocysts were graded according to the Gardner criteria. For biopsy, blastocysts with an expansion grade ≥3 and a trophectoderm deemed suitable for safe cell removal (typically grade C or better) were selected on Day 5, 6, or 7. Early blastocysts (expansion ≤2) were not biopsied. For outcome analysis, a “high-quality blastocyst” was defined as having a Gardner score of AA, AB, BA, or BB, reflecting high developmental potential. Cycles yielding no biopsy-suitable blastocysts were excluded from ploidy outcome analysis but included in analyses of pre-biopsy embryological outcomes. Embryologists performing all morphological assessments were blinded to paternal clinical information, including age, as embryo laboratory identifiers were linked solely to maternal records.

### Embryo biopsy

On day 5 or 6, 5–10 cells were biopsied from the trophectoderm of blastocyst-stage embryos. Whole genome amplification was performed using the multiple annealing and looping-based amplification cycles (MALBAC) method, following the manufacturer's standard protocol (Yikon Genomics Inc, China). The biopsied cells were carefully transferred into 5 μL of Lysis Reaction Mix in a polymerase chain reaction (PCR) tube. Cell lysis was achieved through heating, followed by sequential amplification.

### Chromosomal copy number variations by nextgeneration sequencing and validation

Chromosomal copy number variation (CNV) analysis was performed according to established methods (13). Following product purification and library preparation, whole genome amplification products were sequenced using the Proton Life DA8600 platform with single-end 40 bp reads. Sequencing parameters were optimized to achieve a minimum depth of 2× coverage, generating between 2 and 5 million raw reads per sample for PGT-A. Each embryo underwent comprehensive genome-wide CNV profiling to accurately determine its ploidy status (euploid or aneuploid).

### Statistical analysis

Continuous variables were expressed as mean ± standard deviation (SD) and analyzed using either the two-tailed unpaired Student's *t*-test (for normally distributed data) or the Mann–Whitney *U*-test (for non-normally distributed data). Categorical variables were presented as counts and percentages, with comparisons made using the chi-square test. Spearman's test was carried out to study the correlation of DFI and different paternal age groups. All statistical analyses were performed using SPSS software (version 22.0; SPSS Inc., Chicago, IL, USA), with statistical significance defined as *P* < 0.05.

Binary logistic regression analyses were conducted to examine the association between paternal age groups and three primary outcomes: high-quality blastocyst formation, clinical pregnancy, and live birth. Male age was categorized into three groups: <35 years, 35–39 years, and ≥40 years (reference group). The dependent variables were defined as follows: high-quality blastocysts, 1 = presence, 0 = absence; clinical pregnancy, 1 = achieved, 0 = not achieved, live birth: 1 = achieved, 0 = not achieved; paternal age group, 2 = paternal age <35–39 years, 1 = paternal age <35 years, 0 = paternal age ≥40 years. Model adjustments were made for the following continuous covariates: maternal age, DFI, sperm concentration, sperm motility, and the number of oocytes retrieved. Total motile sperm count (TMSC) was considered but excluded due to collinearity with its constituent parameters. The data were shown as odds ratios (ORs) and 95% confidence intervals (CIs).

A sensitivity analysis was performed to further address potential residual confounding by maternal age. Specifically, the cohort for analyzing the formation of high-quality blastocysts and frozen embryo transfer (FET) outcomes was restricted to women aged ≤32 years. Within this restricted sub-cohort, multivariate logistic regression models were re-run to assess the robustness of the primary findings regarding the associations of paternal age and DFI with embryological outcomes (e.g., high-quality blastocyst formation) and clinical outcomes (clinical pregnancy, live birth). Due to the substantially reduced sample size in this stringent sub-cohort, which limited statistical power for clinical endpoints, the detailed results of the clinical outcome analyses are presented in the Supplementary Material for transparency, while the embryological outcomes are discussed in the main text.

A *post-hoc* power calculation was conducted to quantify the statistical power for detecting differences in clinical pregnancy and live birth rates between paternal age groups, given the limited sample size in the ≥40 years group. The calculation focused on the comparison between the combined paternal age <40 years group (*n* = 210 FET cycles) and the ≥40 years group (*n* = 49 FET cycles). The analysis was performed for a two-tailed test of two independent proportions with an alpha level of 0.05, using G*Power software 3.1.

## Results

### General characteristics of the study population

**Figure 1:**
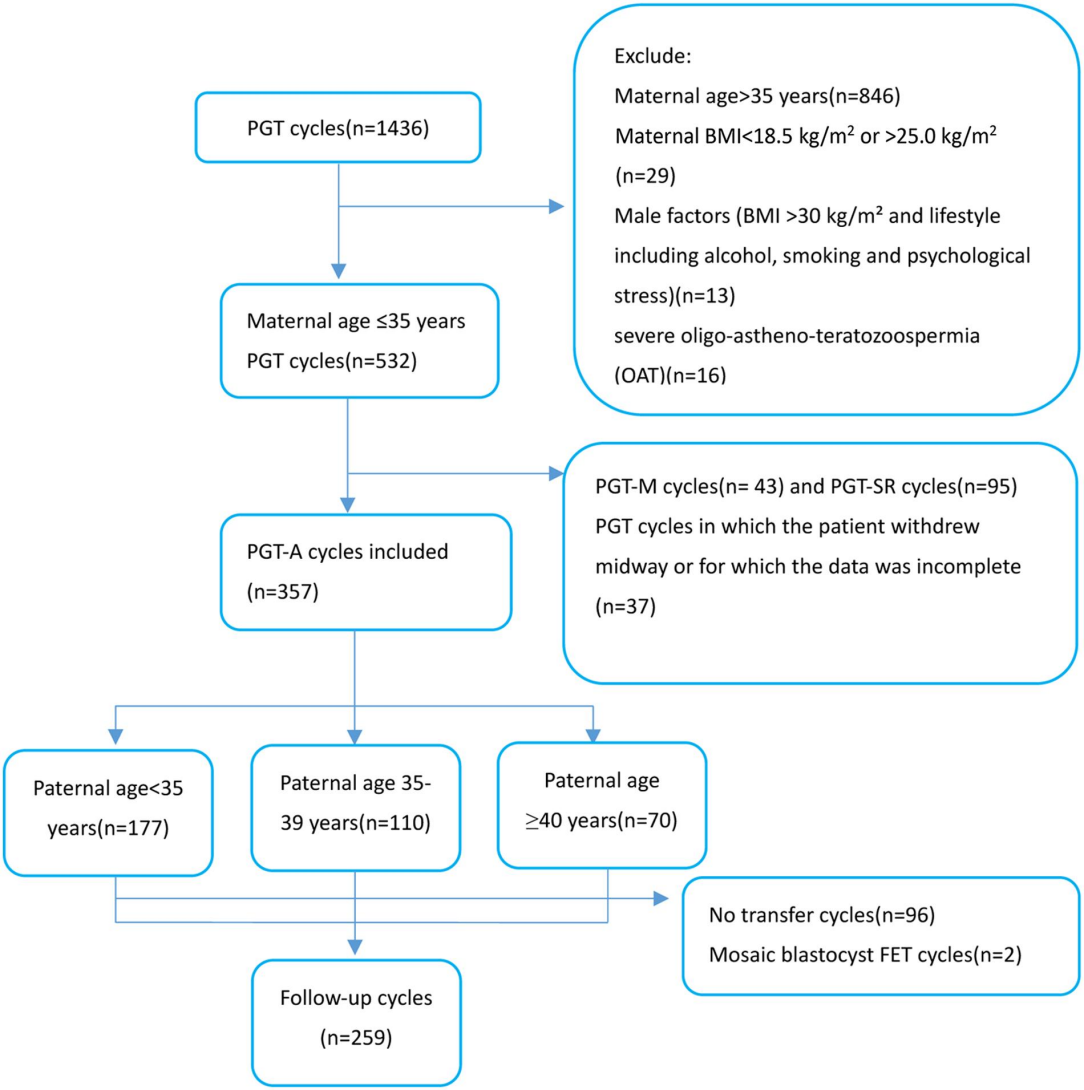
Flow diagram of all patients included in this study. BMI, body mass index; IVF, *in vitro* fertilization; PGT-A, preimplantation genetic testing for aneuploidy; PGT-M, preimplantation genetic testing for monogenic; PGT-SR, preimplantation genetic testing for structural rearrangements; FET, frozen-thawed embryo transfer.

A total of 1,436 couples undergoing their first PGT cycle were initially enrolled. After applying the exclusion criteria, 846 cycles with maternal age >35 years, 29 with maternal BMI <18.5 or >25.0 kg/m^2^, 13 with relevant paternal factors (BMI >30 kg/m^2^ and/or lifestyle factors), and 16 with severe oligo-astheno-teratozoospermia were excluded. This resulted in an analytical cohort of 532 PGT cycles from women aged ≤35 years (Figure 1). Subsequent exclusions comprised cycles involving preimplantation genetic testing for monogenic disorders (PGT-M, *n* = 43) or for chromosomal structural rearrangements (PGT-SR, *n* = 95), as well as cycles where patients withdrew or had incomplete data (*n* = 37). Consequently, 357 cycles designated for PGT-A were included for the primary analysis. These cycles were stratified into three groups based on paternal age at the time of oocyte retrieval: <35 years (*n* = 177), 35–39 years (*n* = 110), and ≥40 years (*n* = 70). Furthermore, after excluding 96 cycles that did not proceed to embryo transfer and 2 cycles involving mosaic blastocyst transfers, follow-up data were available for 259 first FET cycles.

The baseline characteristics of the patients are presented in Table 1. The age range was 25–35 years for women and 24–54 years for men. As anticipated, there was a statistically significant difference in paternal age across the three groups (all *P* < 0.05), given that paternal age served as the primary criterion for group classification. However, our analysis revealed a statistically significant increase in the average maternal age for men aged 35–39 years (33.8 ± 1.2) and those aged ≥40 years (33.1 ± 1.9) when compared to their counterparts under 35 years of age (31.7 ± 2.0; both *P* < 0.05). In the context of infertility patterns, no significant differences were observed in the rates of other infertility patterns. Baseline serum hormone levels, including follicle-stimulating hormone (FSH) and luteinizing hormone (LH), showed no significant variations across the groups (both *P* > 0.05). And there were no statistically significant differences in BMI between the paternal and maternal sides across the three groups. Additionally, while the number of oocytes retrieved was numerically lower in the ≥40 years group compared to the younger age groups, these differences did not reach statistical significance (all *P* > 0.05).

**Table 1:** Characteristics of the PGT-A cycle in the different paternal age groups.

Characteristic	Paternal age group		
	<35-year-old	35–39-year-old	≥40-year-old
PGT-A cycles (*n*)	177	110	70
Paternal age(year), mean ± s.d.	32.0 ± 2.0	36.3 ± 1.4**^a<0.001^**	43.9 ± 4.4**^bc(both<0.001)^**
Maternal age(year), mean ± s.d.	31.7 ± 2.0	33.8 ± 1.2**^a<0.001^**	33.1 ± 1.9**^c<0.001^**
Pattern of infertility			
Primary infertility, % (*n*/total)	27.7 (49/177)	35.5 (39/110)	30.0 (21/70)
Secondary infertility, % (*n*/total)	72.3 (128/177)	64.5 (71/110)	70.0 (49/70)
Recurrent Miscarriage, % (*n*/total)	14.7 (26/177)	10.9 (12/110)	15.7 (11/70)
History of IVF failure % (*n*/total)	4.0 (7/177)	7.3 (8/110)	10.0 (7/70)
Female pelvic and tubal factors, % (*n*/total)	4.0 (7/177)	5.5 (6/110)	8.6 (6/70)
Asthenozoospermia, % (*n*/total)	37.3 (66/177)	36.4 (40/110)	47.1 (33/70)
Oligozoospermia, % (*n*/total)	9.0 (16/177)	7.3 (8/110)	8.6 (6/70)
Maternal BMI (kg m^−2^), mean ± s.d.	21.8 ± 2.9	22.0 ± 2.9	21.4 ± 2.6
Paternal BMI (kg m^−2^), mean ± s.d.	25.7 ± 4.0	25.7 ± 3.5	25.2 ± 1.9
Basal FSH (mIU ml^−1^), mean ± s.d.	8.2 ± 3.4	7.3 ± 2.9	8.5 ± 2.9
Basal LH (mIU ml^−1^), mean ± s.d.	4.4 ± 2.4	4.2 ± 2.0	4.4 ± 1.4
Retrieved oocytes (*n*), mean ± s.d.	14.6 ± 9.0	14.6 ± 8.6	13.0 ± 8.0
Metaphase II oocytes (*n*), mean ± s.d.	11.8 ± 7.1	11.8 ± 7.2	11.0 ± 5.6

a,c The results are significantly different (*P* < 0.001) compared with those of the <35-year-old group; ^b^ the results are significantly different (*P* < 0.001) compared with those of the 35–39-year-old group. For paternal age, all corresponding *P* values for ^a,b^, and ^c^ were <0.001. For maternal age, both the *P* values for ^b^, and ^c^ were <0.001. All included patients were confirmed via medical record review to be non-smokers and without a history of excessive alcohol use. IVF, *in vitro* fertilization; s.d., standard deviation; BMI, body mass index; FSH, follicle-stimulating hormone; LH, luteinizing hormone; PGT-A, preimplantation genetics testing for aneuploidy.

### Sperm characteristics of the cohort

As presented in Table 2, the sperm parameters were within the normal ranges as defined by the 5th edition of the WHO criteria (2010) with minor modifications. Notably, males aged ≥40 years demonstrated statistically significant declines in both sperm concentration (*P* = 0.027) and total sperm count (*P* = 0.008), along with elevated DFI levels (*P* = 0.001), when compared to those in the <35 years age group. Similarly, men aged >40 years showed statistically significant reductions in total sperm count (*P* = 0.043) and increased DFI levels (*P* = 0.016) relative to males aged 35–39 years. However, no significant differences were observed among the three paternal age groups in terms of semen volume, progressive motility (PR), or normal morphology (all *P* > 0.05). Additionally, Spearman correlation analysis showed a positive correlation between paternal age groups and DFI (*P* < 0.0001, *R* = 0.461, Table 3).

**Table 2:** Semen parameters of in PGT-A cycles in the different paternal age groups.

Semen parameter	Paternal age groups		
	<35-year-old	35–39-year-old	≥40-year-old
PGT-A cycles (*n*)	177	110	70
Volume (ml), mean ± s.d.	2.8 ± 1.6	2.6 ± 1.2	2.3 ± 0.6
Concentration (×10^6^ ml^−1^), mean ± s.d.	53.3 ± 19.8	51.7 ± 22.2	45.3 ± 17.7**^c^** **^=^** **^0.027^**
Total sperm count/ejaculated (×10^6^), mean ± s.d.	147.1 ± 92.1	135 ± 84.5	103.6 ± 42.9**^bc(b^** **^=^** **^0.043; c^** **^=^** **^0.008)^**
Progressive motility (%), mean ± s.d.	38.1 ± 14.0	38.5 ± 12.7	34.3 ± 12.8
Normal morphology (%), mean ± s.d.	4.4 ± 1.5	4.5 ± 1.5	4.1 ± 1.2
DFI, mean ± s.d.	15.7 ± 9.3	18.1 ± 9.5	25.2 ± 16.8**^bc(b^** **^=^** **^0.016;c^** **^<^** **^0.001)^**

PGT-A, preimplantation genetics testing for aneuploidie; DFI, DNA fragmentation index; s.d., standard deviation.

b The results are significantly different (*P* < 0.05) compared with those of the 35–39-year-old group; ^c^The results are significantly different (*P* < 0.01) compared with those of the <35-year-old group. For concentration, the *P* value of 0.027 for ^c^. For total sperm count/ejaculated, the *P* values of 0.043 for ^b^, 0.008 for ^c^. For DFI, the *P* values of 0.016 for ^b^, less than 0.001 for ^c^.

**Table 3:** Spearman correlation analysis between DFI and different paternal age groups.

Semen parameter	Paternal age groups	
DFI	Coefficient (*R*)	0.461^a^
	*P*-value	0.000

Spearman correlation was performed to study the relationship between DFI and different paternal age groups.

a The correlation is significant at level 0.01 (two-tailed).

### Embryo quality and clinical outcomes in PGT-A cycles

A total of 357 cycles were analyzed in this study, categorized into three groups based on paternal age: 177 cycles in the <35-year-old group, 110 cycles in the 35–39-year-old group, and 70 cycles in the ≥40-year-old group (Table 4). Comparative analysis across these paternal age groups demonstrated no statistically significant differences in normal fertilization rate, high-quality embryo rate, euploid blastocyst rate or mosaicism rate (all *P* > 0.05). In contrast, the rates of blastocyst formation (57.3% vs. 68.6%; 57.3% vs. 67.2%, all *P* < 0.001) and high-quality blastocyst formation (33.2% vs. 41.3%; 33.2% vs. 40.2%, all *P* < 0.01) were markedly lower in the ≥40-year-old group than in the <35-year-old group and the 35–39-year-old group.

**Table 4:** *In vitro* fertilization laboratory data of three different paternal age groups.

Laboratory data	Paternal age groups		
	<35-year-old	35–39-year-old	≥40-year-old
PGT-A cycles(*n*)	177	110	70
Oocytes retrieved (*n*)	2,579	1,608	910
Metaphase II oocytes (*n*)	2,090	1,296	769
Normal fertilization (2PN) rate, % (*n*/total)	78.7 (1,645/2,090)	77.6 (1,006/1,296)	79.5 (611/769)
High-quality embryo rate, % (*n*/total)	52.0 (856/1,645)	52.1 (524/1,006)	53.2 (325/611)
Blastocyst formation rate, % (*n*/total)	68.6 (1,129/1,645)	67.2 (676/1,006)	57.3 (350/611)**^bc(b^** **^<^** **^0.001, c^** **^<^** **^0.001)^**
High-quality blastocyst rate, % (*n*/total)	41.3 (679/1,645)	40.2 (404/1,006)	33.2 (203/611)**^bc(b^** **^=^** **^0.005, c^** **^<^** **^0.001)^**
Blastocyst for biopsy	1,078	668	289
Euploid blastocysts rate, % (*n*/total)	56.9 (613/1,078)	52.7 (352/668)	51.6 (149/289)
Mosaicism rate, % (*n*/total)	17.4 (188/1,078)	18.1 (121/668)	13.8 (40/289)

**^b^**The results are significantly different (*P* < 0.01) compared with those of the 35–39-year-old group; ^**c**^The results are significantly different (*P* < 0.05) compared with those of the <35-year-old group. For blastocyst formation rate, all corresponding *P* values for **^b^** and **^c^** were less than 0.001. For high-quality blastocyst rate, the *P* values of 0.005 for **^b^**, less than 0.001 for ^**c**^.

Of the 357 couples, 96 did not proceed to embryo transfer following the initial oocyte retrieval. After the exclusion of two mosaic blastocyst transfer cycles, a total of 259 FET cycles were analyzed. These were categorized by paternal age as follows: 132 cycles in the <35-year-old group, 78 cycles in the 35–39-year-old group, and 49 cycles in the ≥40-year-old group (Table 5). Blastocysts with a morphological grading of higher than 4CC were eligible for PGT-A to identify euploid blastocysts for transfer. Single blastocyst transfer was performed in all first FET cycles. Analysis revealed lower clinical pregnancy and live birth rates in the ≥40-year age group compared to both the <35-year (49.0% vs. 64.4%; 44.9% vs. 58.3%) and 35–39-year groups (49.0% vs. 62.8%; 44.9% vs. 55.1%). However, these differences did not reach statistical significance. Similarly, miscarriage rates showed no significant differences across groups. Furthermore, preterm birth and multiple pregnancy rates demonstrated no significant variation among the paternal age groups (all *P* > 0.05). Ectopic pregnancy occurred exclusively in the 35–39-year-old group.

**Table 5:** Clinical outcomes in three different paternal age groups after frozen-thawed euploid blastocyst transfer treatment.

Laboratory data	Paternal age groups		
	<35-year-old	35–39-year-old	≥40-year-old
FET cycles (*n*)	132	78	49
Transferred blastocysts (*n*)	132	78	49
Clinical pregnancy, % (*n*/total)	64.4 (85/132)	62.8 (49/80)	49.0 (24/49)
Miscarriage, % (*n*/total)	9.4 (8/85)	6.4 (5/78)	8.3 (2/24)
Live birth, % (*n*/total)	58.3 (77/132)	55.1 (43/78)	44.9 (22/49)
Preterm birth, % (*n*/total)	3.9 (3/77)	4.7 (2/43)	9.1 (2/22)
Multiple pregnancy, % (*n*/total)	4.7 (4/85)	2.0 (1/49)	4.2 (1/24)
Ectopic pregnancy, % (*n*/total)	0 (0/85)	2.0 (1/49)	0 (0/24)
Birth defects, % (*n*/total)	0 (0/85)	0 (0/50)	0 (0/24)

FET, frozen-thawed embryo transfer; s.d, standard deviation.

### Binary logistic regression analysis of high-quality blastocyst formation, clinical pregnancy, and live birth

To examine the influence of paternal age on high-quality blastocyst formation, clinical pregnancy and live birth outcomes, we performed multivariate regression analyses, incorporating paternal age groups, DFI, maternal age, sperm concentration, sperm motility, and number of oocytes retrieved as independent variables, with high-quality blastocyst, clinical pregnancy and live birth serving as the dependent variables, respectively.

The results indicated that DFI was an independent factor influencing both high-quality blastocyst formation and clinical pregnancy, where higher DFI was associated with a significantly reduced likelihood of high-quality blastocyst formation (OR = 0.987, *P* = 0.046) and clinical pregnancy (OR = 0.961, *P* = 0.036). And the number of oocytes retrieved was an independent influencing factor for high-quality blastocyst formation (OR = 1.019, *P* = 0.002). In contrast, paternal age, maternal age, sperm concentration and sperm motility did not exhibit a statistically significant impact on either high-quality blastocyst formation, clinical pregnancy or live birth outcomes (Table 6).

**Table 6:** Logistic regression analysis of paternal age for high-quality blastocyst formation, clinical pregnancy and live birth.

Item	High-quality blastocyst formation (*n* = 2,155)			Clinical pregnancy(*n* = 259)			Live birth (*n* = 259)		
	OR	95% CI	*P*	OR	95% CI	*P*	OR	95% CI	*P*
Paternal age groups			0.352			0.168			0.256
Paternal age groups (1)	1.263	0.753–2.117	0.376	2.694	0.937–7.750	0.066	2.300	0.808–6.542	0.119
Paternal age groups (2)	1.451	0.845–2.491	0.177	2.499	0.751–8.318	0.135	2.343	0.716–7.668	0.159
DFI	0.987	0.975–1.000	0.046^a^	0.961	0.926–0.997	0.036^a^	0.971	0.937–1.007	0.111
Sperm concentration	0.999	0.996–1.001	0.303	0.998	0.990–1.006	0.673	0.999	0.991–1.006	0.743
Sperm motility	0.998	0.989–1.007	0.678	1.024	0.990–1.059	0.169	1.014	0.982–1.047	0.396
Maternal age	1.038	0.975–1.106	0.242	1.063	0.883–1.279	0.520	1.128	0.943–1.350	0.187
Number of oocytes retrieved	1.019	1.007–1.031	0.002^b^	0.978	0.944–1.013	0.213	0.990	0.956–1.025	0.572
constant	0.329	–	0.303	0.123	–	0.498	0.016	–	0.170

OR, odds ratio; CI, confidence interval; DFI, DNA fragmentation index. The reference category for paternal age is ≥40 years.

a The results indicated that DFI was an independent influencing factor both for high-quality blastocyst formation and clinical pregnancy (both *P* > 0.05)

b The result indicated that number of oocytes retrieved was an independent influencing factor for high-quality blastocyst formation (*P* < 0.01).

To control for maternal age confounding, a sensitivity analysis was performed within the subgroup of women aged ≤32 years. In this highly restricted cohort, DFI remained a significant, independent negative predictor of high-quality blastocyst formation (adjusted odds ratio, aOR = 0.980, *P* = 0.009). Compared to the paternal age ≥40 years group, the paternal age 35–39 years group was associated with significantly higher odds of forming high-quality blastocysts (aOR = 2.542, *P* = 0.025)(Table 7).

**Table 7:** Logistic regression analysis of high-quality blastocyst formation in the restricted cohort (maternal age ≤32 years).

Item	High-quality blastocyst formation(*n* = 736)		
	Adjusted OR (aOR)	95% CI	*P*
Paternal age groups			0.059
Paternal age groups (1)	1.542	0.773–3.078	0.219
Paternal age groups (2)	2.542	1.122–5.760	0.025^a^
DFI	0.980	0.965–0.995	0.009^a^
Sperm concentration	1.006	0.998–1.014	0.157
Sperm motility	0.989	0.977–1.001	0.067
Maternal age	1.000	0.909–1.101	0.998
Number of oocytes retrieved	1.015	0.997–1.035	0.110
constant	1.320		0.857

OR, odds ratio; DFI, DNA fragmentation index. The reference category for paternal age is ≥40 years.

a Are present the statistically significant results (*P* < 0.05).

Importantly, maternal age itself showed no significant association within this model (*P* = 0.998). These findings demonstrate that the associations of paternal age and sperm DFI with embryo quality persist even after extreme restriction of maternal age.

### Birth and newborn outcomes after FET treatment

FET cycles were calculated as the number of enrolled patients who underwent the first FET treatment. The analysis included a total of 148 live births (136 singletons and 12 twins) resulting from 158 pregnancy cycles. As detailed in Table 8, the delivery and newborn outcomes following FET treatment were comparable across the paternal age groups. Specifically, no statistically significant differences were observed in the birth length of singletons. Although singleton birth weight was numerically lower in the ≥40 years group, this difference did not reach statistical significance. Apart from one preterm birth in the 35–39-year-old group, no other abnormalities in birth weight or length were noted. However, the limited sample size precludes definitive conclusions regarding the influence of advanced paternal age (≥40 years), warranting further investigation with larger cohorts.

**Table 8:** Birth and newborn characteristics after frozen-thawed embryo transfer treatment.

Variable	Paternal age groups		
	<35-year-old	35–39- year-old	≥40-year-old
Pregnancy cycles (*n*)	85	49	24
Live births (*n*)	81	44	23
Singleton babies (*n*)	73	42	21
Male babies (*n*)	38	26	9
Female babies (*n*)	35	16	12
Birth weight of singletons (g), mean ± s.d	3,441.1 ± 430.2	3,557.4 ± 396.6	3,366.7 ± 523.7
Birth length of singletons (cm), mean ± s.d	51.0 ± 2.2	51.2 ± 2.3	51.2 ± 2.7
Twins babies (*n*)	8	2	2
Male babies (*n*)	5	0	2
Female babies (*n*)	3	2	0
Birth weight of twins(g), mean ± s.d	2,571.9 ± 535.2	1,225.0 ± 0.5	2,500.0 ± 10.0
Birth length of twins (cm), mean ± s.d	44.1 ± 6.4	34.5 ± 0.5	48.5 ± 0.5

s.d., standard deviation.

## Discussion

Currently, significant attention focuses on the impact of APA on fertilization rates and ICSI outcomes. Multiple studies indicate that older paternal age negatively affects semen parameters, fertility potential, pregnancy outcomes, and ICSI success (14, 15). This study specifically assessed the potential influence of paternal age differences on sperm parameters, embryo quality, embryo ploidy, and clinical outcomes in PGT-A cycles, while controlling for maternal age and the euploidy status of transferred embryos. We suggested that the paternal age ≥40 years group was associated with significantly lower sperm concentration and total count, alongside a higher DFI, consistent with prior literature (16–18).

While the negative effects of APA on laboratory and clinical outcomes are documented, the evidence remains conflicting, largely due to confounding factors such as maternal age, the presence of severe male factor infertility, and the efficacy of interventions like ICSI (19). In fact, there is not even consensus regarding the definition or age cut-off of APA. Studies utilizing donor oocytes suggest that paternal age >50 years may adversely affect pregnancy outcomes and blastocyst formation rates (20). However, these findings primarily stem from studies involving ICSI cycles for oligozoospermic males (21). A meta-analysis reported a slight but significant linear decrease in live birth rate with increasing paternal age in egg donation cycles, with a similar non-significant trend for clinical pregnancy rate (22). Importantly, analyses of many oocyte donation cycles fail to adequately address the age of the maternal recipients as an uncontrolled variable (23), contributing to inconclusive results. Recent research indicates that when ICSI is performed (regardless of male factor infertility), male age may not impact live birth rate per oocyte retrieval or per embryo transfer, potentially because ICSI overcomes age-related sperm defects (19). APA (>44 years) was reported to be associated with slower blastocyst development and poorer morphological quality in PGT-A cycles (24). A recent meta-analysis demonstrated that euploid embryo transfers (ET) in APA couples yielded comparable LBRs to those in non-APA couples, with no significant difference in miscarriage rate (MR) per clinical pregnancy (25). These findings align with two additional studies examining paternal age as a continuous variable in relation to euploid ET outcomes (26, 27), which similarly reported no association between APA and LBR or MR.

In our study, although all enrolled female patients were ≤35 years old, maternal age was significantly higher in groups where the paternal partner was ≥40 or 35–39 years old compared to the <35 years group, indicating an age-pairing tendency. Importantly, a sensitivity analysis restricted to women ≤32 years provided further clarity. Within this sub-cohort of minimal maternal age variability, a clearer gradient related to paternal age emerged. The paternal age group 35–39 years was significantly higher odds of forming high-quality blastocysts compared to the ≥40 years group. The negative association between DFI and blastocyst quality remained robust. This suggests that the pronounced influence of maternal age in broader cohorts may obscure more subtle correlations with paternal factors. The significance of high-quality blastocysts in the 35–39 vs. ≥40 comparison but not in the <35 vs. ≥40 comparison, possibly reflecting an accelerated decline in sperm genomic integrity around the late thirties. Collectively, our data support a model in which advanced paternal age (≥40 years) is associated with diminished embryo quality largely through the pathway of increased sperm DNA damage, an effect that becomes salient when maternal factors are optimally controlled. The clinical outcome models within our restricted maternal age (≤32 years) sensitivity analysis were underpowered due to the substantially reduced sample size (Supplementary Table S1). Therefore, the observed associations with clinical pregnancy and live birth in this sub-cohort should be interpreted as preliminary and require confirmation in larger, dedicated studies.

Research indicates that elevated DFI negatively impacts conception rates and embryonic development. High levels of DNA damage can disrupt embryogenesis and trigger apoptotic pathways (28, 29). While the underlying causes of elevated DFI remain incompletely characterized, current evidence implicates defects in DNA repair during spermatogenesis, compounded by secondary damage from oxidative stress, inflammatory mediators, metabolic byproducts, or elevated temperatures. The clinical significance of DFI remains controversial. Some studies propose that oocyte repair mechanisms can compensate for sperm DNA damage (30), while others suggest DFI exerts detrimental effects, particularly in IVF where natural sperm selection occurs. In contrast, ICSI bypasses this selection, relying on visual assessment of motility and morphology. This may inadvertently introduce sperm with DNA defects, potentially correlates with blastocyst development (29). Notably, DFI levels exceeding 15% correlate with abnormal embryo morphokinetics, cleavage patterns, and reduced embryo quality (29). Conflicting findings persist, with some studies reporting minimal DFI effects in ICSI (31), while others demonstrate significant negative correlations between DFI and blastocyst formation, quality, and usable blastocyst rates (29). Furthermore, DFI has been shown to impair implantation without affecting fertilization rates in ICSI cycles (32). Our findings support the hypothesis that APA is associated with increased DFI, potentially compromising blastocyst quality and clinical pregnancy success. However, to quantify the concern regarding the limited sample size in the paternal age ≥40 years group, we performed a *post-hoc* power calculation for the comparison of clinical outcomes between the <40 years (*n* = 210) and ≥40 years (*n* = 49) groups (Supplementary Table S2). These results suggested that our study was substantially underpowered (conventional threshold: 80%) to detect the observed, clinically meaningful differences in pregnancy and live birth rates. This also suggests that our conclusions should be validated in a larger PGT-A population. Emerging evidence links APA to an increased incidence of both numerical and structural chromosomal abnormalities in sperm (33). Concurrently, age-related changes in sperm epigenetic markers, particularly DNA methylation patterns, are consistently associated with male infertility (34). Collectively, these observations underscore the significant impact of sperm DNA integrity and epigenetic regulation on ART outcomes.

The effect of paternal age on embryonic aneuploidy rates remains debated (35–38). While one study observed a decreased euploid rate after age 40 (10), another found no significant difference in euploidy rates between embryos from male partners aged ≤38 vs. >38 years after adjusting for female age (39). The latter finding is consistent with our results, which showed no statistically significant variation in blastocyst euploidy rates across different paternal age groups. Moreover, a meta-analysis indicated that embryo biopsy day and maternal age—but not paternal age—were associated with the occurrence of mosaicism in embryos (40). And our data demonstrated no statistically significant differences in mosaicism rates among the paternal age groups analyzed. Therefore, based on current evidence, we tend to conclude that advanced paternal age does not exert a notable effect on embryonic euploidy or mosaicism.

Similar to maternal age exceeding 35 years, paternal age of 40 years is considered a critical threshold, beyond which adverse reproductive outcomes and potential offspring health concerns may emerge. While APA does not constitute a primary risk factor for infertility in euploid embryo transfers, it demonstrates a significant association with elevated DFI. Current evidence suggests that APA may correlate with increased *de novo* mutations and epigenetic alterations in offspring (41). A recent study revealed that positive selection during spermatogenesis elevates the risk of known disease-causing mutations by 2–3 fold. This leads to a scenario where 3%–5% of sperm in middle-aged and older men carry a pathogenic exomic mutation (42). These findings not only illuminate the dynamics of germline selection but also indicate that advanced paternal age confers a greater disease risk to offspring than previously recognized. The full consequences of which may only be elucidated through long-term postnatal follow-up studies. It should be noted that this study only conducted statistics on the weight and length of newborns, and long-term follow-up was not statistically analyzed.

In summary, this study demonstrates that advancing paternal age significantly elevates sperm DFI, and increased DFI negatively correlates with high-quality blastocyst rate, which may influence clinical pregnancy rate. In this study, we did not observe any impact of advanced paternal age on the euploidy of embryos. However, once a euploid embryo is achieved and transferred, APA itself does not appear to negatively impact pregnancy outcomes. A notable limitation of this study is that the relationship between increased paternal age and adverse pregnancy outcomes in patients who did not yield any euploid blastocysts remains unevaluated, constrained by the design of our study cohort. Furthermore, the limited number of FET cycles, particularly for patients with paternal age ≥40 years, resulted in insufficient statistical power to robustly establish the associations between paternal age, sperm DFI, and clinical endpoints (clinical pregnancy and live birth). Therefore, these specific relationships necessitate validation in future studies with larger, dedicated sample sizes.

## Data Availability

The raw data supporting the conclusions of this article will be made available by the authors, without undue reservation.
