# Supplementary material for: Impact of advanced paternal age on reproductive outcomes in preimplantation genetic testing cycles of young female: a retrospective cohort study
Source: Front Reprod Health. 2026 Jan 23;7:1750842. doi: 10.3389/frph.2025.1750842 (PMC12876146; doi:10.3389/frph.2025.1750842)
Supplement: Supplementary file 1 [file Table1.docx]

Supplementary

**Table S1：Logistic regression analysis of clinical pregnancy and live birth in the restricted cohort (Maternal Age ≤32 Years).**

| *Item* | Clinical pregnancy(n=102) | | | | | | Live birth (n=102) | | | | |  |
| --- | --- | --- | --- | --- | --- | --- | --- | --- | --- | --- | --- | --- |
|  | Adjusted OR (aOR) | *95%CI* | | *P* | | Adjusted OR (aOR) | | *95%CI* | | | *P* |  |
| Paternal age groups |  |  | 1.000 | |  | | | |  | 1.000 | | |
| Paternal age groups(1) | 1993871419.287 | 0.000 | 0.999 | | 2094929946.060 | | | | 0.000 | 0.999 | | |
| Paternal age groups(2) | 0.537 | 0.000 | 1.000 | | 0.632 | | | | 0.000 | 1.000 | | |
| DFI | 0.981 | 0.925-0.981 | 0.518 | | 0.992 | | | | 0.938-1.049 | 0.772 | | |
| Sperm concentration | 1.011 | 0.994-1.029 | 0.214 | | 1.009 | | | | 0.992-1.025 | 0.302 | | |
| Sperm motility | 1.012 | 0.965-1.063 | 0.617 | | 0.990 | | | | 0.946-1.036 | 0.668 | | |
| Maternal age | 0.986 | 0.692-1.406 | 0.940 | | 1.055 | | | | 0.764-1.456 | 0.745 | | |
| Number of oocytes retrieved | 0.966 | 0.890-1.049 | 0.409 | | 1.015 | | | | 0.940-1.097 | 0.700 | | |
| constant | 0.000 |  | 0.999 | | 0.000 | | | |  | 0.998 | | |

The reference category for paternal age is ≥40 years. OR, odds ratio; DFI, DNA fragmentation index.
