# Supplementary material for: Impact of advanced paternal age on reproductive outcomes in preimplantation genetic testing cycles of young female: a retrospective cohort study
Source: Front Reprod Health. 2026 Jan 23;7:1750842. doi: 10.3389/frph.2025.1750842 (PMC12876146; doi:10.3389/frph.2025.1750842)
Supplement: Supplementary file 2 [file Table2.docx]

**Table S2. Post-Hoc Power Analysis for clinical outcomes by paternal age group**

| *Parameter* | Group (<40 years) | Group (≥40 years) | Effect size (Cohen's h) | Achieved power (1-β)(%) |
| --- | --- | --- | --- | --- |
| FET cycles(n) | 210 | 49 | - | - |
| Observed clinical pregnancy rate(%) | 63.3 (133/210) | 49.0 (24/49) | 0.291 | 47 |
| Observed live birth rate(%) | 56.7 (/119/210) | 44.9 (22/49) | 0.238 | 42 |

Post-hoc power was calculated for two-group comparisons (paternal age <40 vs. ≥40 years) of clinical pregnancy and live birth rates. Calculations were performed using G*Power 3.1 for a two-tailed test of two independent proportions, with an alpha (α) level of 0.05. Effect size is expressed as Cohen‘s h.
