# Supplementary material for: Impact of advanced paternal age on reproductive outcomes in preimplantation genetic testing cycles of young female: a retrospective cohort study
Source: Front Reprod Health. 2026 Jan 23;7:1750842. doi: 10.3389/frph.2025.1750842 (PMC12876146; doi:10.3389/frph.2025.1750842)
Supplement: Supplementary file 3 [file Table3.docx]

**Table S3 The rationale for the inclusion of all covariates considered**

| Suggested variable | action taken & location in revision | primary rationale |
| --- | --- | --- |
| Sperm total motility | Added. Included as a continuous covariate in all Binary logistic regression models. (Tables 6, S1). | Its inclusion strengthens the model by accounting for basic sperm function. |
| Number of oocytes retrieved | Added. Included as a continuous covariate in all Binary logistic regression models (Tables 6, S1). | A major determinant of the embryo developmental potential. |
| Sperm total motile count (TMSC) | Not added. | It is mathematically derived from sperm concentration and total motility, which are already in the model. Inclusion would cause severe multicollinearity and obscuring the independent effect of each parameter. |
| Number of blastocysts available for biopsy | Not added. | It is considered a downstream outcome or mediator of early embryo development rather than a true confounder. Including it would lead to over-adjustment bias by statistically controlling for part of the causal pathway we aim to study. |
| Lifestyle factors (e.g., smoking, alcohol) | Not added as model covariates. | These were pre-specified exclusion criteria for the entire study cohort. All included patients were confirmed via medical record review to be non-smokers and without a history of excessive alcohol use, as stated in the Methods. |
